# Correction: Monitoring independence in daily life activities after trauma in humanitarian settings: Item reduction and assessment of content validity of the Activity Independence Measure-Trauma (AIM-T)

**DOI:** 10.1371/journal.pgph.0003002

**Published:** 2024-02-27

**Authors:** Bérangère Gohy, Christina H. Opava, Johan von Schreeb, Rafael Van den Bergh, Aude Brus, Abed El Hamid Qaradaya, Jean-Marie Mafuko, Omar Al-Abbasi, Sophia Cherestal, Livia Fernandes, Andre Da Silva Frois, Eric Weerts, The AIM-T Study Group, Nina Brodin

There are errors in the Funding and Competing Interests statements. The correct statements are:

**Funding:** Enhancing Learning and Research in Humanitarian Action (ELRHA) funded the research coordination (BG) and research activity for this study as part of their Health in Humanitarian Crises (R2HC) programme (No.32398), funded by the UK Foreign, Commonwealth and Development Office (FCDO), Wellcome, and the Department of Health and Social Care (DHSC) through the National Institute for Health Research (NIHR) (https://www.elrha.org/programme/research-for-health-in-humanitarian-crises/). Médecins Sans Frontières (MSF) provided support in the form of salaries for RVdB, AEHQ, JMM, OAA, SC, LF, and ADSF. The specific roles of these authors are articulated in the ‘author contributions’ section. MSF also provided support in the form of salaries for AIM-T Study Group members NH, ECG, EN, MF, VM, JVH, AA, and CM. The funders otherwise had no role in study design, data collection and analysis, decision to publish, or preparation of the manuscript.

**Competing Interests:** The authors have read the journal’s policy and have the following competing interests to declare: Médecins Sans Frontières (MSF) provided support in the form of salaries for RVdB, AEHQ, JMM, OAA, SC, LF, and ADSF, as well as for AIM-T Study Group members NH, ECG, EN, MF, VM, JVH, AA, and CM. This does not alter our adherence to *PLOS Global Public Health* policies on sharing data and materials. There are no patents, products in development, or marketed products associated with this research to declare.
