# Supplementary material for: Monitoring independence in daily life activities after trauma in humanitarian settings: Item reduction and assessment of content validity of the Activity Independence Measure-Trauma (AIM-T)
Source: PLOS Glob Public Health. 2022 Dec 14;2(12):e0001334. doi: 10.1371/journal.pgph.0001334 (PMC10021394; doi:10.1371/journal.pgph.0001334)
Supplement: S1 Fig — (DOCX) [file pgph.0001334.s001.docx]

**S1 Fig. Activity Independence Measure-Trauma, first version (AIM-T_1_)**

| **Lower limb Sub score** |  | **Upper limb Sub score** |  |
| --- | --- | --- | --- |
| **Locomotion** : |  | **Hygiene :** |  |
| Walk around (less than 50 meters) | 1 2 3 4 5 | Wash your back | 1 2 3 4 5 |
| Walk around (over 50 meters) | 1 2 3 4 5 | **Dexterity :** |  |
| Go up stairs | 1 2 3 4 5 | Grab cup of tea | 1 2 3 4 5 |
| Go down stairs | 1 2 3 4 5 | Open a jar | 1 2 3 4 5 |
| **Transfers :** |  | Opposition thumb-5th finger | 1 2 3 4 5 |
| Sit up | 1 2 3 4 5 | Grab pen | 1 2 3 4 5 |
| Stand up | 1 2 3 4 5 | Eating | 1 2 3 4 5 |
| Sit down | 1 2 3 4 5 | **Upper Limb activities :** |  |
| Lie down | 1 2 3 4 5 | Carry object overhead | 1 2 3 4 5 |
| **Toilet :** |  | Comb hair | 1 2 3 4 5 |
| Full squat | 1 2 3 4 5 | Put on pants | 1 2 3 4 5 |
| **Pray :** |  | Put on shirt | 1 2 3 4 5 |
| Kneeling (sitting) | 1 2 3 4 5 |  |  |
|  |  |  |  |
| **TOTAL Lower Limb sub score** | **____/ 50** | **TOTAL Upper Limb sub score** | **____/ 50** |
|  |  |  |  |
| 1= Total assistance; 2 = Assistance (human support); 3 = Modified independence (use of device); 4 = Independence with difficulties; 5 = Independence | | | |
