# Supplementary material for: Monitoring independence in daily life activities after trauma in humanitarian settings: Item reduction and assessment of content validity of the Activity Independence Measure-Trauma (AIM-T)
Source: PLOS Glob Public Health. 2022 Dec 14;2(12):e0001334. doi: 10.1371/journal.pgph.0001334 (PMC10021394; doi:10.1371/journal.pgph.0001334)
Supplement: S2 Fig — (DOCX) [file pgph.0001334.s002.docx]

**S2 Fig. Activity Independence Measure-Trauma, third version (AIM-T_3_)**

| **Changing & Maintaining position** |  |  |  |
| --- | --- | --- | --- |
| Roll over | 0 1 2 3 4 5 |  |  |
| Sit up and remain seated for 10 seconds | 0 1 2 3 4 5 |  |  |
| **TOTAL Core score** | _______/ **10** |  |  |
|  |  |  |  |
| **Changing & Maintaining position** |  | **Fine hand use** |  |
| Stand up and remain standing for 10 seconds | 0 1 2 3 4 5 | Pick up small object and manipulate | 0 1 2 3 4 5 |
| Kneel down and stand up | 0 1 2 3 4 5 | **Hand and arm use** |  |
|  |  | Open a jar/bottle | 0 1 2 3 4 5 |
| **Walking & Moving** |  | Reach lower back and grasp clothes | 0 1 2 3 4 5 |
| Walk/Move around 14m | 0 1 2 3 4 5 | Reach face and neck | 0 1 2 3 4 5 |
| Timed 10m walk/move around* | 0 1 2 3 4 5 |  |  |
| Climb up and down 10 steps | 0 1 2 3 4 5 | **Lifting and carrying objects** |  |
|  |  | Lift and carry 5kg above shoulder level | 0 1 2 3 4 5 |
|  |  |  |  |
| **TOTAL Lower Limb score** | **______ / 25** | **TOTAL Upper Limb score** | **______ / 25** |

0= totally dependent; 1 = dependent on human support; 2 = dependent on equipment/environment modification with difficulties; 3 = dependent on equipment/environment modification without difficulties; 4 = independent with difficulties; 5 = totally independent

*For timed 10m walk/move: **if >12.5 seconds**: 0= totally dependent; 1 = dependent on human support/equipment; **if ≤12.5 seconds**: 2 = dependent on equipment/environment modification with difficulties; 3 = dependent on equipment/environment modification without difficulties; 4 = independent with difficulties; 5 = totally independent
