# Supplementary material for: Monitoring independence in daily life activities after trauma in humanitarian settings: Item reduction and assessment of content validity of the Activity Independence Measure-Trauma (AIM-T)
Source: PLOS Glob Public Health. 2022 Dec 14;2(12):e0001334. doi: 10.1371/journal.pgph.0001334 (PMC10021394; doi:10.1371/journal.pgph.0001334)
Supplement: Attachment — Submitted filename: PGPH-D-22-01425_Response to reviewers.docx [file pgph.0001334.s003.docx]

Bérangère Gohy

Handicap International

44, Rue de l’arbre bénit

1050 Brussels, Belgium

0032-498-708530

[b.gohy@hi.org](mailto:b.gohy@hi.org)

November 8^th^ 2022

Dear editor,

We would like to thank you for having considered our manuscript PGPH-D-22-01425
“Monitoring independence in daily life activities after trauma in humanitarian settings: Item reduction and assessment of content validity of the Activity Independence Measure-Trauma (AIM-T)” for its publication in PLOS Global Public Health.

We have carefully reviewed the different comments and have provided an answer for each of the points raised by the editor and reviewer(s). We hope that our manuscript now meets the criteria for publication in PLOS Global Public Health.

Thank you for your consideration,

Sincerely,

On behalf of all the co-authors,

Bérangère Gohy,

Handicap International

|  | Response to point raised | Change(s) made |
| --- | --- | --- |
| 1. Please review your reference list to ensure that it is complete and correct. If you have cited papers that have been retracted, please include the rationale for doing so in the manuscript text, or remove these references and replace them with relevant current references. Any changes to the reference list should be mentioned in the rebuttal letter that accompanies your revised manuscript. If you need to cite a retracted article, indicate the article’s retracted status in the References list and also include a citation and full reference for the retraction notice. | Thank you for identifying those discrepancies, some references information had been mistyped. | The reference 5 (line 463-465) has been updated for its authors and year of publication, and reference 42 (line 567-569) for its authors and title. |
| 2. Please send a completed 'Competing Interests' statement, including any COIs declared by your co-authors. If you have no competing interests to declare, please state "The authors have declared that no competing interests exist". Otherwise please declare all competing interests beginning with the statement "I have read the journal's policy and the authors of this manuscript have the following competing interests:" | Thank you for identifying this. We confirm that there are no competing interests to declare. | We have now updated the competing interests, as required. |
| 3. We do not publish any copyright or trademark symbols that usually accompany proprietary names, eg ©, ®, or ™  (e.g. next to drug or reagent names). Please remove all instances of trademark/copyright symbols throughout the text, including ® on page 33. | Thank you for your comment. | We have deleted the trademark symbol in reference 42 (previously 41). |
| While revising your submission, please upload your figure files to the Preflight Analysis and Conversion Engine (PACE) digital diagnostic tool, <https://pacev2.apexcovantage.com/>. PACE helps ensure that figures meet PLOS requirements. To use PACE, you must first register as a user. Registration is free. Then, login and navigate to the UPLOAD tab, where you will find detailed instructions on how to use the tool. If you encounter any issues or have any questions when using PACE, please email PLOS at [figures@plos.org](mailto:figures@plos.org). Please note that Supporting Information files do not need this step. | We do not have any figures as part of the main manuscript, only in the supporting information files. We have therefore not used PACE when revising the submission. |  |
| Reviewer #1 | | |
| I am of the opinion from what is presented in the manuscript in all sections, that the authors have presented a well written report of their work. The abstract is clear, the introduction and methodological approach appears good. | Thank you for your supportive comment. |  |
| Reviewer #2 | | |
| In my view, the study described in the manuscript addresses a pragmatic and relevant topic, which is the development and validation of a tool to uniformly measure functioning after trauma in humanitarian contexts. A plus is an attention the authors had in trying to overwhelm some limitations of the existing measures (i.e. some activities may be present in some cultures while not in others) and to propose a tool that might be applied by the different stakeholders in humanitarian contexts. | Thank you for your detailed critical review of our manuscript. |  |
| Overall, the study consisted of two steps: (i) reduction of the number of items of the original version of a previously designed scoring system (AIM-T1); (ii) assessment of the content validity of the new scoring system with reduced items (AIM-T2) and development of a new, validated tool (AIM -T3).  The abstract clearly introduces the topic and the objectives of the study and well summarises the adopted methodology as well as the main results.  The introduction is overall well written and logically defines the problem that the study aims to solve.  The methodology is described in detail and follows a clear logical order of the item reduction and content validity assessment steps. The authors are aware that some requirements of the COSMIN methodology for assessing content validity were not followed, but clearly state this in the limitations (lines 374-377) and motivate why the content validity can be anyway supported (lines 377-379). | Thank you. |  |
| • Is some more information about the location (i.e. head injury, pelvic injury, thoracic injury…), severity (Injury Severity Score?) and mechanism (i.e., violence, car accident..) of the injury available? If yes, a table could be added referring to lines 248-255 and also added to the groups in table 1. | Thank you for your question and suggestion for an additional Table. Some information requested is however not available as medical information came from routine data only. The ISS is for example not routinely calculated in those projects. Regarding the location of injury, we have only data at the level of lower, upper and trunk injury, as presented.  The cause of injury was not collected for the interviews’ participants. | We have added a Table 1 describing the characteristics of the patients included in the item reduction analysis, and adapted the referring lines accordingly.  We have also renumbered all the tables following the addition of table 1. |
| • Lines 135-137: how many AIM-T scores were available for IPD admission and discharge and OPD admission and discharge? | Thank you for your relevant question. | We have now added that extra information in the results section, line 255-256.  For clarity we have also added “admission to” and “discharge from” in the title of Table 3. |
| • Line 223: it’s not clear to me why a 15% threshold was chosen for the addition of an item: are there references about this percentage? | To our knowledge there is no gold standard regarding the threshold for addition of new item. We have set the 15% threshold based on what has been done by other authors in the same field (i.e. Hoffman et al. 2016). | We have now cited the reference (38) in the Methods section, line 223. |
| • Lines 284-287: why toileting and walking long distances, although mentioned by >15% of the participants were not included in the final version of the questionnaire? Also, I don’t see them in table 3. | As mentioned in the analysis section, lines 223-225 :“Aspects of feasibility and appropriateness of observing the suggested activities were also considered when revising the AIM-T_2_ items”.  More specifically for the two items:  Toileting:  The item “squatting” had a low I-CVI in terms of appropriateness. This was most often related to the fact that “squatting” is associated with “toileting” and was therefore considered by participants as a inappropriate activity to perform in front of somebody else. Therefore, even though “toileting” was frequently mentioned by participants, we decided not to include it as such in the AIM-T3, but rather use mobility activities, namely “kneeling down and stand up” and “reach lower back and grasp clothes” to cover some of its content.  Walking long distances:  As discussed in lines 348 to 359, it did not seem feasible to observe the patients walking long distances (i.e. kilometers). Since it was very frequently mentioned by participants, we have identified a proxy (i.e. Timed 10 meter walk). Indeed the self-chosen speed during this test has been reported as associated with ability to ambulate in community. | We have now provided more information on the reasons for not including both items as such, in line 299-301  We hope that this addition, on top of methods on line 223 and discussion on lines 339-347 for “toileting” and lines 348 to 359 for “walking long distances” clarifies this point. |
| • Lines 298-303: why the scoring system was expanded by one level (5◊6)? I read “based on participant’s description…and feedback..”, but what does this mean? | The scoring system was indeed expanded to add the scoring level “2= dependent on equipment/environment modification with difficulties”.  This addition was based on the concept analysis, where “difficulties” were mentioned by 32 participants (either referring to the needed time “slowly”, the quality of movement “limping”, “using compensation mechanisms” “not in a straight line”, pain during movement, safety issues such as “fear of falling” or “unsteady”, or more globally the ease/smoothness/difficulties experienced). | We have now added precisions on this aspect in lines 287-289, and 303-305. |
| • Some of the AIM-T3 items reported in the last column of table 3 are slightly different from those reported in the final version of the questionnaire (i.e. sit up and remain seated for 10 seconds vs sit up (supine edges); stand up and remain standing for 10 seconds vs stand up (sit to stand); lift and carry 5 kg above shoulder level vs lift 5 kb above shoulder level…). Which is the correct version? | Thank you for identifying these discrepancies. | We have now harmonized the names of the items in both the Supplement 2 and in the Table 3, as well as in line 351 and 356. |
| • How was the 14 meters distance chosen? Is this the correspondent of walking long distances or is it something in between short and long distances? | As explained in line 362-365, the 14 metre distance was chosen to allow the assessment of both walking activities at the same time (i.e. “14m walk/move around”, as well as “Timed 10m walk/move around”). Indeed, one of the protocols for the Timed 10m walk test administration is to ask the patient to walk 14 metres, timing the middle 10 metres. | We have added some clarification lines 357-359, as well as a reference to the test protocol used by other authors. |
| The discussion is well-written and clearly summarises the relevance of the AIM-T3, as well as its limits and pros. The authors are aware that this tool will need further validation for wider use in different scenarios and clearly state this in the discussion. | Thank you for your encouraging comments. |  |
